# New Results on Small and Dim Infrared Target Detection

**DOI:** 10.3390/s21227746

**Published:** 2021-11-21

**Authors:** Hao Wang, Zehao Zhao, Chiman Kwan, Geqiang Zhou, Yaohong Chen

**Affiliations:** 1 Xi’an Institute of Optics and Precision Mechanics, Chinese Academy of Sciences, Xi’an 710119, China; wanghao@opt.ac.cn (H.W.); zhaozehao19@ucas.ac.cn (Z.Z.); 2 University of Chinese Academy of Sciences, Beijing 100049, China; 3 Signal Processing, Inc., Rockville, MD 20850, USA; chiman.kwan@signalpro.net; 4 Astronaut Selection and Training Center, Beijing 100094, China; zhgeqiang@163.com

**Keywords:** IR target detection, real-time detection, imaging processing

## Abstract

Real-time small infrared (IR) target detection is critical to the performance of the situational awareness system in high-altitude aircraft. However, current IR target detection systems are generally hardware-unfriendly and have difficulty in achieving a robust performance in datasets with clouds occupying a large proportion of the image background. In this paper, we present new results by using an efficient method that extracts the candidate targets in the pre-processing stage and fuses the local scale, blob-based contrast map and gradient map in the detection stage. We also developed mid-wave infrared (MWIR) and long-wave infrared (LWIR) cameras for data collection experiments and algorithm evaluations. Experimental results using both publicly available datasets and image sequences acquired by our cameras clearly demonstrated that the proposed method achieves high detection accuracy with the mean AUC being at least 22.3% higher than comparable methods, and the computational cost beating the other methods by a large margin.

## 1. Introduction

High-altitude aircraft has great potential in early warning and detection, space offense and defense, and electronic countermeasures, which become increasingly significant in a modern battlefield. The ability to detect long-distance dim and small targets is essential for high-altitude aircraft to obtain real-time battlefield information and perceive threats during missions. A high-performance infrared (IR) detection system for dim and small targets is an indispensable instrument onboard high-altitude aircraft due to its key advantages including long detection range and all-weather surveillance capability [1].

The imaging and detection of dim and small infrared targets in high-altitude scenarios have distinctive characteristics. First, the target size is typically less than 3 × 3, or even only 1 pixel due to long imaging distance. The optical path attenuation and large field of view (FOV) result in low signal-to-noise ratio (SNR) of the output image without any contour and textual features, making it difficult to detect by learning based methods [2,3,4]. Second, the background of the image is dominated by clouds, which are vulnerable to the negative impacts caused by irradiation and reflection of sunlight or moonlight, and these impacts further increase the difficulty of detection. Third, with the rapid development of infrared detector technology and performance, the latest IR imaging systems have shown a trend of “three highs” (high resolution, high frame rate, and high dynamic range). Consequently, these imaging systems put forward extremely high requirements on the computational efficiency of data processing methods, and a large number of conventional algorithms cannot meet the real-time requirements [5]. Finally, due to the difficulty of data acquisition for research and experiments, there are only a few high-performance IR detection techniques for dim and small targets in high-altitude scenarios.

Due to the large FOV of the situational awareness system, multiple targets with different scales may appear within the effective detection range, and the scale of the target changes as the distance between detection system and target changes. Moreover, fast flying targets have large displacement between adjacent frames, while target flying along the optical axis has small displacement between adjacent frames, making it more difficult to establish a robust sequence-based detection method. Therefore, the single-frame detection algorithm is more suitable for such applications.

Previous works on single-frame detection can be generally divided into three categories: filtering-based methods, algorithms based on image data structure, and local feature-based approaches. The filtering-based methods typically use spatial or frequency domain filters to suppress the background or enhance the targets and then further distinguish the targets from the background. Deshpande et al. [6] proposed to suppress the clutters by max-mean/max-medium filter. Cao et al. [7] improved the two-dimensional least mean square (TDLMS) filter and incorporated neighborhood analysis and data fusion. Bai et al. [8] detected the small target by gradually decreasing the structure element in Top-hat filter. Bae et al. [9] first introduced the bilateral filter to small IR target detection. However, all of the aforementioned algorithms cannot achieve promising results in complex backgrounds.

Detection methods based on image data structure include low-rank sparse decomposition, tensor recovery, robust principal component analysis (RPCA), etc. Gao et al. [10] convert the infrared patch-image (IPI) model into an optimization problem based on sparse matrix. Zhang et al. proposed to detect the small IR target by non-convex rank approximation minimization (NRAM) [11] and partial sum of the tensor nuclear norm (PSTNN) [12]. Dai et al. [13] used a priori information to adaptively assign weights to each column based on the IPI model and proposed a weighted infrared patch image (WIPI) model. Huang et al. [14] proposed to detect targets by density peaks searching and maximum-gray region growing. Qin et al. [15] developed an algorithm based on facet kernel and random walker (FKRW). However, the aforementioned methods have difficulty in achieving robust detection performance in complex and changing scenarios.

The key idea of the local feature-based approaches is to detect targets by exploiting the different characteristics between the targets and the background. Kim et al. [16] and Wang et al. [17] first introduced the Laplacian of Gaussian (LoG) filter and the difference of Gaussian (DoG) filter for IR target detection, respectively. However, the above blob detection filters are susceptible to spot-like backgrounds and blinking pixels. Chen et al. [18] developed local contrast measure (LCM), which enhanced the target significantly but has difficulty in distinguishing targets and strong clutters. Wei et al. [19] developed a multiscale patch-based contrast measurement (MPCM) to improve the processing speed of LCM. Deng et al. [20] proposed a weighted local difference measure (WLCM) to enhance the target and suppress the background simultaneously. Han et al. [21] developed a relative local contrast measure (RLCM). Although a large number of methods are derived based on the idea of LCM, single feature detection has limited performance on low SNR dataset. Consequently, a combination of multiple features such as local intensity and gradient (LIG) [22] properties has become a new trend.

In the application of long-distance imaging systems, previous work generally has the problems of rapidly degrading performance under complex cloud backgrounds and difficulty in meeting the real-time application requirements. The filtering-based methods including TDLMS and Top-hat filter cannot handle complex scenarios [21]. The computational cost of the methods based on image data structure such as IPI and WIPI increases exponentially with the image resolution, while with the NRAM and PSTNN methods it is easy to regard some clutter in complex scenes as the target. Methods based on local features, especially local contrast methods, typically have difficulties in distinguishing the targets from the clutter, especially hot blinking-pixels with strong gray value.

Inspired by [16,17,22], a few of significant features can be used for the small IR target detection. For one thing, the small targets (less than 3 × 3), which exhibit pop-out behavior and blob-like morphology, can be remarkably enhanced by the blob detection filter. For another, there are huge local contrast and gradient differences between the target pixels and background pixels. Consequently, the pop-out behavior and blob-like morphology enable efficient candidate extraction, while the local contrast and gradient properties have advantages indistinguishing real targets from clutters effectively. A recent paper by us [23] exploited the above IR target characteristics and proposed a new detection algorithm for small and dim target detection.

In this paper, we present new results of applying the efficient and effective detection method combining local contrast, blob-like morphology and scale and gradient features in [4]. The key contributions of the proposed method are described as follows:

To further improve the detection performance in real imaging system, the earlier framework for small and dim target detection method in [23] was fine-tuned. Although the computational time is a little more than the earlier version, the background clutter is better suppressed.

To the best of our knowledge, we are the first to develop a high-performance MWIR camera to evaluate the single-pixel target detection performance, which plays a significant role in long range imaging systems.

We present new target detection results using both publicly available datasets and datasets acquired by our own cameras, meaning that the proposed method has promising application value.

The computational cost of our method is still much less than other state-of-the-art approaches.

This paper is organized as follows: Section 2 describes the details of the proposed method. Section 3 summarizes the experimental results and comparison with other methods. Finally, Section 4 concludes the paper with a few future directions.

## 2. Detection Based on the Local Contrast and Gradient Feature

The incorporation of local contrast, blob-like morphology, and scale and gradient features for effective small target detection was proposed in our recent paper [23]. In this section, we include more details of the various steps of target detection. We also fine-tuned our algorithm with more emphasis on background clutter suppression.

**Figure 1:**
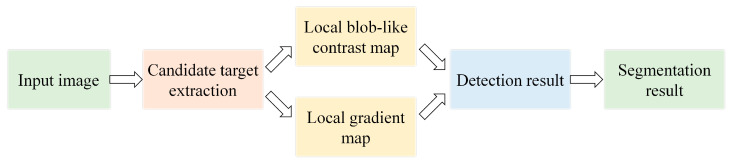
Framework of the proposed IR small target detection method.

Figure 1 illustrates the framework of the proposed method. In the pre-processing stage, we apply a DoG filter to the input image and introduce the cumulative distribution function (CDF) to generate a binarized mask, which contains a small proportion of candidate pixels. The mask enables a fast and accurate overall target detection system. In the detection stage, we calculate the local contrast map by blob-like morphology feature and local gradient map by four quadrant analysis. We also fuse the local contrast map and gradient map. Finally, we accurately extract the targets by performing segmentation.

In the next few subsections, we will describe the details of pre-processing stage, detection procedure, and segmentation.

### 2.1. Pre-Processing and Binary Mask Generation

**Figure 2:**
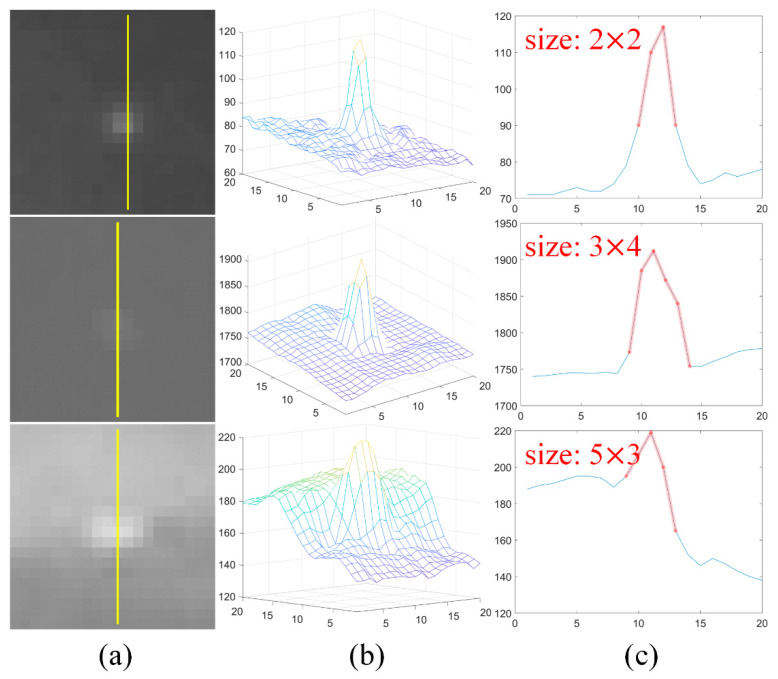
Demonstration of the 3-D and 1-D analysis for small IR targets with different sizes: (**a**) local patches contain targets; (**b**) 3-D mesh; (**c**) 1-D cross-section profile analysis.

Figure 2 demonstrates the 3-D and 1-D analysis for small IR targets with different sizes in the sky-cloud scenarios. It is clear that all of the targets including that on the edge of strong clouds (the third column of the Figure 2a) show the blob-like characteristics in the 3-D mesh (Figure 2b) and 1-D cross-section profile analysis (Figure 2c), meaning that the blob detection filters can be used to enhance the target effectively.

Since the previous application of the Laplacian of Gaussian (LoG) filter [16] and the difference of Gaussian (DoG) filter [17], which are widely used in blob-target detection, we propose to apply the DoG filter to the input image because the DoG is more hardware-friendly than the LoG filter [24]. Let $\sigma_{1}$ and $\sigma_{2}$, which are customer-selected parameters, denote the standard deviations of the Gaussian functions; the DoG filter can be explicitly expressed by

(1) $$DoG(i,j,\sigma_{1},\sigma_{2})=\frac{1}{2\pi \sigma_{1}^{2}}e^{-(i^{2}+j^{2})/2\sigma_{1}^{2}}-\frac{1}{2\pi \sigma_{2}^{2}}e^{-(i^{2}+j^{2})/2\sigma_{2}^{2}},$$

The DoG filter is almost the same as the LoG filter [24], which is one of the most popular blob detectors if $\sigma_{2}/\sigma_{1}=1.6$. Since the single-pixel target shows blob-like morphology due to the atmospheric turbulence and the point spread function (PSF) of optical lens, a single-pixel target affects about 3×3 pixels in the image. Given that the target sizes typically range from 3×3 to 5×5 in our applications and the LoG filter has the highest response with a target diameter of $2\sqrt{2}\sigma$ [25], we set $\sigma_{1}=3/2\sqrt{2}=1.06$ and $\sigma_{2}=1.6\times \sigma_{1}=1.70$, respectively. We also found that a minor adjustment of $\sigma_{1}$ and $\sigma_{2}$ will not significantly affect the results.

**Figure 3:**
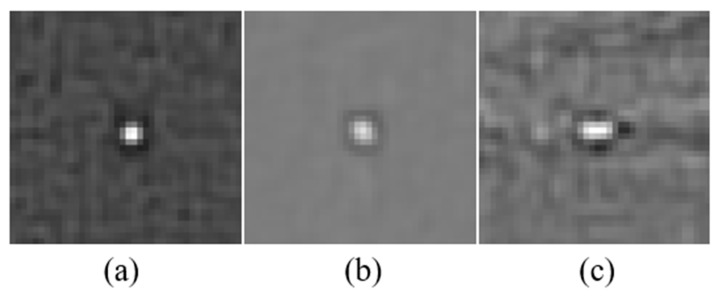
The filtering results of the targets in the Figure 2.

The filtering results of the targets in Figure 2 are shown in Figure 3. Let *I*_D_ represent the filtering result of the DoG filter. There is no doubt that the targets are enhanced remarkably in *I*_D_. However, the DoG filter is susceptible to flickering noise and spot-like backgrounds, which means that high performance detection cannot be obtained by using only the DoG filter. Therefore, we propose to extract candidate target pixels as a binarized mask by calculating the cumulative distribution function (CDF) of *I_D_* and extracting the pixels with the highest intensity. Let the adjustable parameter *p* represent the proportion of candidate pixels; we found that *p* = 0.2% works excellent inour experiments. The pseudocode for the binarized mask generation is illustrated in Algorithm 1. Let *I_M_* denote the binarized mask, all the subsequent processing stages are guided by *I_M_*, meaning that the proposed method has great advantage in computational cost.
**Algorithm 1.** Pseudocode for Binarized Mask Calculation.**Input:** The DoG filtering result $I_{D}$, *p* = 0.2%, gray dynamic range N
**Output:** The binarized mask $I_{M}$
1:$\left[row,col]=size(I_{D}\right)$;2:$I_{M}=zeros(row,col)$;3:**for** i=1:row4:    **for** j=1:col5:       $h(I_{D}(i,j)+1)++$;6:    **end**7:**end**8:P(k)=h(k)/(row×col),   k∈[1,N];9:CDF(k)=P(k)+CDF(k−1);10:$I_{M}(find(CDF(I_{D})>=(1-p))=1$

### 2.2. Local Blob-like Contrast Map

The targets are typically enhanced remarkably by the DoG filter, while the surrounding areas of the targets are drastically suppressed, as shown in Figure 3. Inspired by the characteristics of *I_D_*, we propose to calculate the local blob-like contrast map by

(2) $$I_{C}(i,j)=(I_{D}(i,j)-\mu_{t})\times (\mu_{t}/\mu_{b}),$$

where $\mu_{t}$ and $\mu_{b}$ are the mean intensity of candidate pixels in the target and surrounding region. The contrast map takes into account both intensity difference and ratio difference between the local target and the surrounding region.

**Figure 4:**
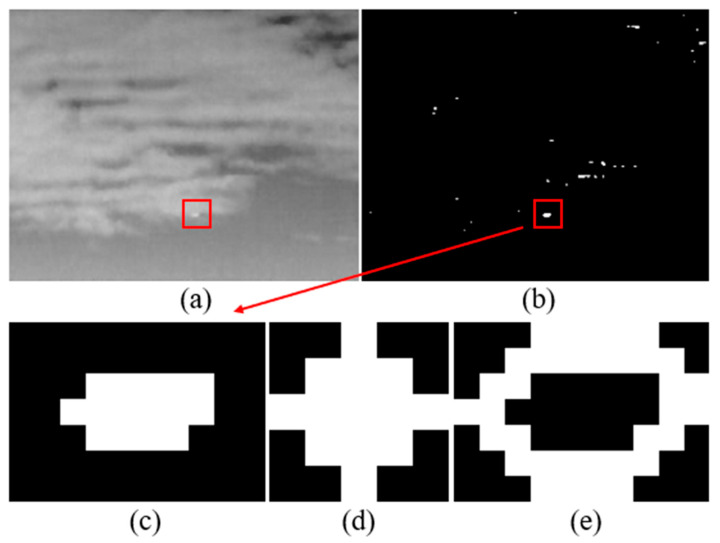
Illustrations of (**a**) input image, (**b**) binarized mask, (**c**) local operation region, (**d**) binarized filter kernel when *r* = 2, and (**e**) the surrounding region represented by the mask.

Here, we should state explicitly that the local mean intensity calculation is guided by connected components in the binarized mask. We find all the connected non-zero regions in the mask and define a surrounding region, which is represented by the convolution result of the local operation region and a binarized filter kernel. Let *L* denote a local operation region, which is a rectangle containing the non-zero regions in the mask, and the binarized filter kernel is defined as follows:

(3) $$w(i,j)=if(\sqrt{{\left(i-c_{row}\right)}^{2}+{\left(j-c_{col}\right)}^{2}}<=r,1,0),$$

where $\left(c_{row},c_{col}\right)$ is the center coordinate of the filter kernel, (i,j) represents the pixel location in the kernel, *r* is a customer-selected parameter, and 2 is a proper value that has been demonstrated to work well in our experiments. The input image and the binarized mask are shown in Figure 4a,b, respectively, and Figure 4c represents the candidate target region. Here we regard the combination of the non-zero pixels in the convolution result of the candidate target region and binarized filter kernel w as the surrounding region, which is illustrated in Figure 4e. It should be noted that even if the clutters such as cloud edge may be denoted by the mask with large scale, the parameters of the DoG filter determine that a small target with a size ranging from 3×3 to 5×5 will achieve a higher enhancement than these clutters, as shown in Figure 4a,b.

### 2.3. Local Gradient Map

Since the detection method based on a single feature makes it difficult to maintain reliable detection performance in various scenarios, we proposed to improve the detection performance by using the fusion of the local contrast and gradient. In the first step, we detect all the pixels in the mask with non-zero values and generate an adaptive scale for each region. We apply all-ones matrix *O_m_* to each candidate pixel, with *m* ranging from 2 to the maximum morphological size of the connected region, which consists of non-zeros pixel clusters. The adaptive scale factor *s* of each region is calculated according to the convolution result of all-ones matrix and connected region in the mask, and the scale for the region is assigned to *m* when the maximum value of the convolution is greater than m(m−1). In order to facilitate the subsequent step, we propose to assign the scale factor 3 to the isolated pixels and clusters with a size less than 3 and adjust the scale factor to an even value by adding 1 if the scale factor is an odd integer.

**Figure 5:**
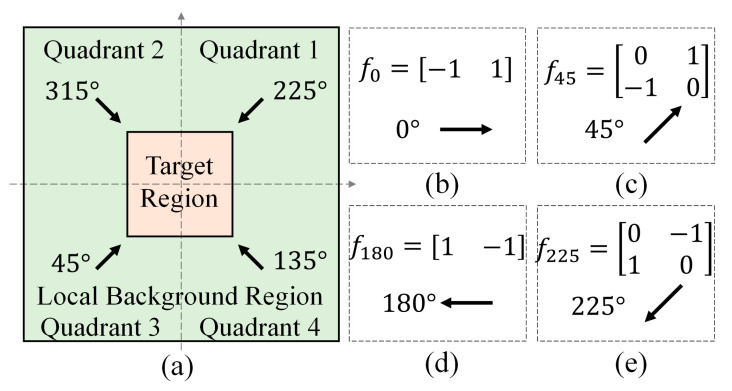
(**a**) Illustration for the operation region for the gradient analysis; (**b**–**e**) gradient filters for different directions.

Inspired by the characteristics described in the introduction, the gradient distribution of an infrared target is typically a few higher than the surrounding areas [16]. We propose to divide the operation region into four quadrants and estimate the gradient features for each quadrant by performing different filters, as shown in Figure 5a. Since filtering results of the directions including 180°, 225°, 270° and 315° can be calculated by inverting the results of directions with 0°, 45°, 90° and 135°, we only perform gradient filtering on four directions, as shown in Figure 5b. Let *S_ij_* represent the sum of elements in matrix obtained by gradient filtering, with the value of *i* is 1 to 4 denoting the four quadrants and the value of *j* is 1 to 8 denoting 0° to 315°, respectively. The gradient score of each quadrant can be calculated by

(4) $$G_{Q1}=a\times S_{16}+b\times (S_{15}+S_{17}),$$

(5) $$G_{Q2}=a\times S_{28}+b\times (S_{21}+S_{27}),$$

(6) $$G_{Q3}=a\times S_{32}+b\times (S_{31}+S_{33}),$$

(7) $$G_{Q4}=a\times S_{44}+b\times (S_{43}+S_{45}),$$

**Figure 6:**
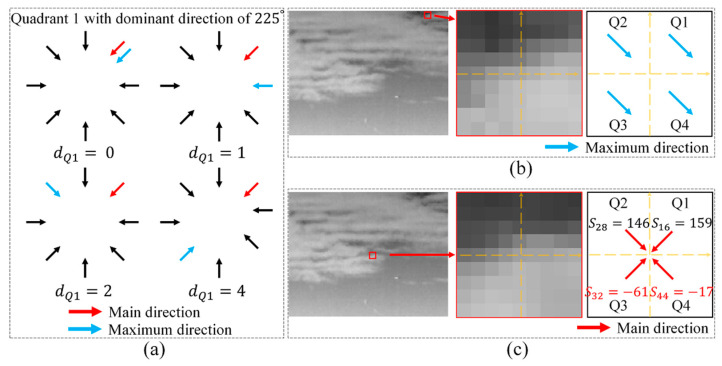
(**a**) Illustration of index distance calculation for quadrant 1 in different situations; (**b**) example of the four quadrants having the same maximum gradient directions; (**c**) example of dominant direction of two quadrants having a negative gradient score.

Both *a* and *b* in Equation (4) to Equation (7) are adjustable parameters and subject to *a* + 2*b* = 1. We set *a* = 0.5 and *b* = 0.25 in our experiments because 225°, 315°, 45° and 135° represent the dominant directions of quadrant 1 to 4, respectively. Let $\mu_{GQ}$ denote the mean gradient feature value of the four quadrants, the intensity of each candidate pixel can be calculated by

(8) $$I_{G}(i,j)=\frac{\mu_{GQ}}{\left(1+p_{1}\right)}\times p_{2},$$

where and $p_{2}$ are penalty factors for the suppression of the strong clutter. The penalty factor $p_{1}$ is defined by

(9) $$p_{1}=\sum d_{Qi},\;\;\;i\in [1,4],\;$$

where $d_{Qi}$ denotes the index distance between the index of the dominant direction and the index with the maximum gradient feature value in quadrant 1~4. The parameter $p_{2}$ is a binarized parameter used to suppress strong edge interference, and $p_{2}$ equals 0 when both the four quadrants has the same maximum directions or at least two quadrants obtain negative scores in the main direction because these two situations are highly related to the appearance of the strong edge. Figure 6a illustrates the index distance $p_{1}$ calculation for quadrant 1 with different situations. Figure 6b,c demonstrates the two situations with penalty factor $p_{2}$ equals zero.

### 2.4. Fusion and Segmentation

To generate the detection result, we fuse the local contrast map and the local gradient map by simply multiplying *I_C_* and *I_G_*:

(10) $$I_{out}=I_{C}\times I_{G}.$$

In the segmentation stage, we propose to segment the detection result by threshold [18]:

(11) $$TH=\mu_{O}+k\times \sigma_{O},$$

where $\mu_{O}$ and $\sigma_{O}$ are the mean and standard deviation of the $I_{out}$, and *k* is a constant ranging from 3 to 5. It should be emphasized that the calculation of the $\mu_{O}$ and $\sigma_{O}$ is only performed for the candidate pixels indicated by the mask.

## 3. Experiments and Discussions

### 3.1. Motivation and Preparation for the Experiments

The IR targets in public datasets are typically bigger than 3×3, and it is hard to acquire images with single-pixel or small-scale target, which are quite significant in the application of the long-distance imaging systems. To evaluate the all-aluminum unobscured two-mirror freeform imaging lens [26] and thermal characteristics of the high-performance camera, we developed a 3.7~4.8 μm MWIR camera and a 7.7–9.7 μm LWIR camera with a resolution of 1024×1024 and 512×512, respectively. The focal lengths of MWIR and LWIR cameras are 21 and 75 mm, respectively. Our dual-band camera is also a good platform for a better implementation and evaluation of the proposed method. Since the detailed hardware description is not the focus of this paper, we mainly introduce the test experiments and results. Given that the LWIR camera is not suitable for single-pixel target detection due to the long focal length, we only use the MWIR camera for the experiment.

**Figure 7:**
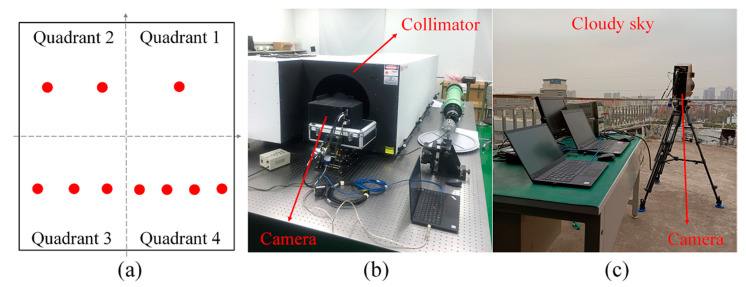
(**a**) Illustration of the target board, (**b**) single-pixel target acquisition, and (**c**) sequence acquisition with cloudy weather.

We made a target board (description in Figure 7a), which contains one, two, three, and four holes in corresponding quadrants, respectively. We then integrated the target board with a blackbody, a collimator with a focal length of 3 m, and our MWIR camera to simulate the imaging and detection performance of single-pixel target, as shown in Figure 7b. We also collected data under different weather conditions, as shown in Figure 7c.

To evaluate the performance of the proposed method, we applied it to both publicly available datasets and images acquired by our cameras. Given that the filtering-based methods have poor detection performance on data sets with complex background, we regard the local feature-based approaches and image data structure-based method including IPI [10], PSTNN [12], FKRW [15], LCM [18], MCPM [19], and LIG [22] as the baseline. All the methods were performed by running them on a laptop with Intel i5-10500 and 16GB RAM and with MATLAB 2020a.

### 3.2. Comparison of Single-Pixel Target Detection Performance

**Figure 8:**
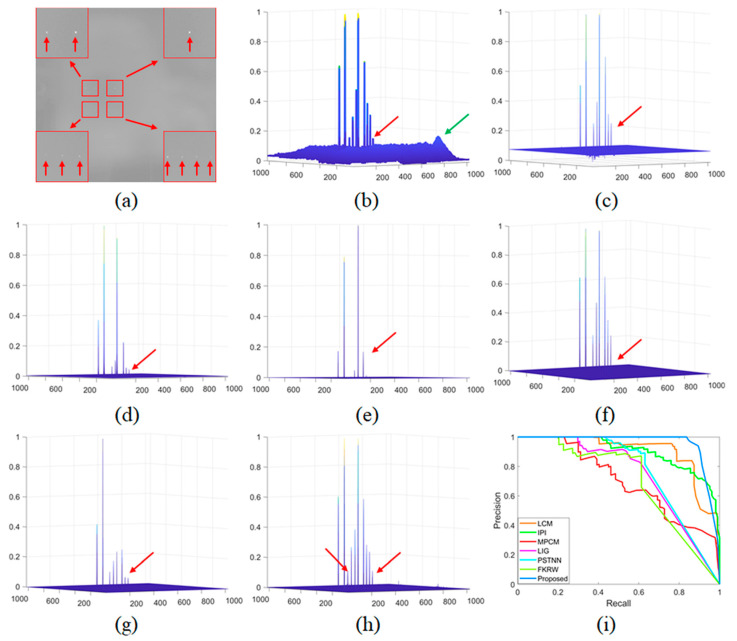
The performance comparison of different methods on single-pixel target detection: (**a**) target image; (**b**–**h**) normalized 3-D mesh obtained by the LCM [18], IPI [10], MCPM [19], LIG [22], PSTNN [12], FKRW [15], and the proposed method; and (**i**) PRC comparison. The red arrows indicate the targets, and the green arrows indicate the noisy clutters.

Since it is quite difficult to acquire images with single-pixel targets, we use target board and collimator to simulate single-pixel target detection. We set the temperature difference between the black body and the ambient temperature to 20 °C and apply a low integration time (1 ms for MWIR camera) to simulate low SNR imaging. It should be emphasized that only MWIR cameras can simulate single-pixel imaging and detection due to its large FOV (41°×41°) and small focal length (*f* = 21 mm). The detection performance comparison of different methods is shown in Figure 8, and the precision–recall curve (PRC) [27] of different methods on single-pixel target detection are shown in the Figure 8h. The target enhancement performance of the LCM is highly related to the target brightness, but the LCM performs poor in terms of clutter suppression. The MPCM improves the LCM’s clutter suppression performance at the cost of the target enhancement ability. The data structure-based methods (IPI, PSTNN, and FKRW) have advantages in clutter suppression, but all of these methods are prone to regard the dim target as the background. It is clear that the proposed method enhances the target and suppresses the background clutters simultaneously with all the targets detected, especially for those challenging targets in quadrant three and quadrant four (shown in Figure 8a).

**Figure 9:**
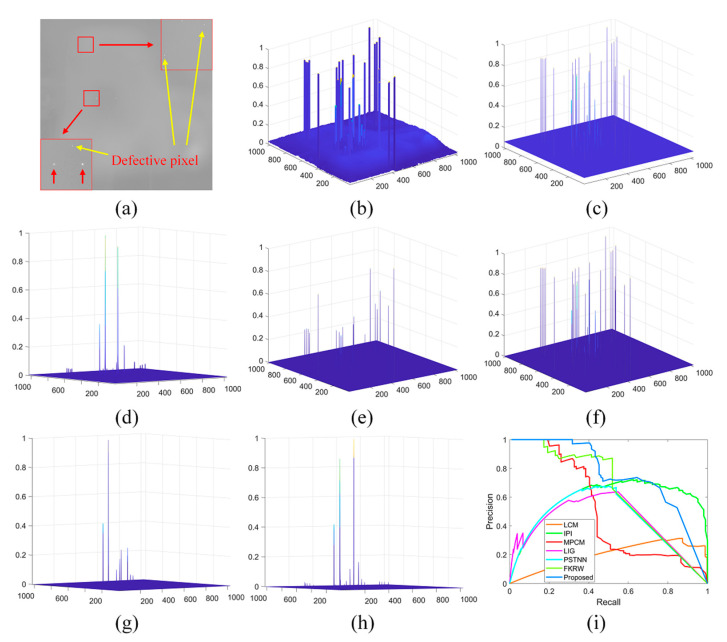
The performance comparison of different methods on single-pixel image with defective pixels: (**a**) target image; (**b**–**h**) normalized 3-D mesh obtained by the LCM [18], IPI [10], MCPM [19], LIG [22], PSTNN [12], FKRW [15], and the proposed method; and (**i**) PRC comparison.

To further compare the robustness of the various methods, we added 20 hot defective pixels with random locations in the single-pixel targets simulation image (Figure 9a) and compared the performances of different methods. The detection performance of all methods has decreased significantly, especially LCM, LIG, and PSTNN have collapsed, which means that these methods are quite sensitive to defective pixel noise. Although our method cannot detect some dim targets, it still maintains an effective suppression of defective pixels.

### 3.3. Comparison of Sequence Detection Performance

The details of the test sequences are described in Table 1. It should be noted that S1 and S2 are open datasets, while S3 and S4 are acquired by our MWIR and LWIR cameras, respectively. Since the proposed method only performs on a few candidate pixels, it is unfair to use the signal-to-clutter ratio gain (SCRG) and the background suppression factor (BSF) as the evaluation metrics [11]. Recent research showed that for detection application on an imbalanced dataset, the precision–recall curve (PRC) has better performance metrics than the receiver operating characteristic (ROC) curve [28,29], so we used the PRC as the evaluation metrics.

**Figure 10:**
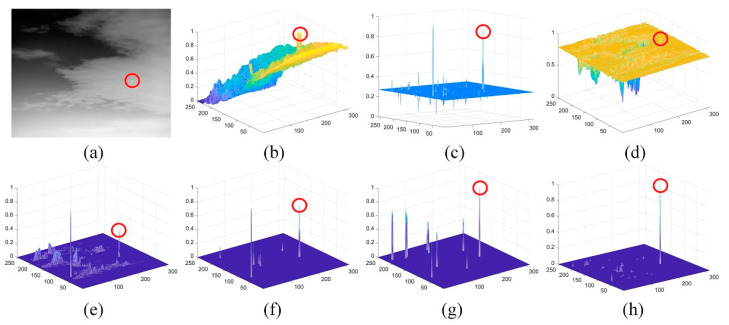
The detection results of different methods on S1: (**a**) the 49th image in S1 and (**b**–**h**) normalized 3-D mesh obtained by the LCM [18], IPI [10], MCPM [19], LIG [22], PSTNN [12], FKRW [15], and the proposed method, respectively.

The detection results of different methods on sequence 1 are shown in Figure 10, and the target is marked by the red circle. The 3-D mesh demonstrated that the proposed method yielded the best detection performance by enhancing the target and suppressing the clutters simultaneously. The IPI, LIG, PSTNN, and FKRW could not distinguish the target from the defective pixel clusters, while the LCM and MPCM failed in suppressing the clutters.

**Figure 11:**
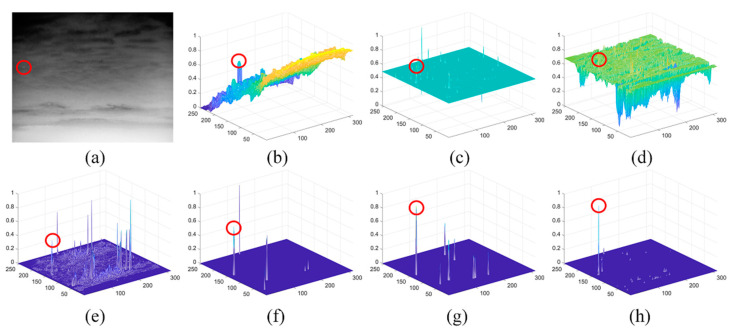
The detection results of different methods on S2: (**a**) the 17th image in S2 and (**b**–**h**) normalized 3-D mesh obtained by the LCM [18], IPI [10], MCPM [19], LIG [22], PSTNN [12], FKRW [15], and the proposed method, respectively.

Figure 11 shows the detection result of a challenging sequence, in which background clutters have a much higher gray value than the target. The LCM was collapsed on S2, while the IPI, MPCM, LIG, and PSTNN cannot distinguish the target from the background because the target has lower intensity than some strong clutters in the background. Although the FKRW can enhance the target effectively, it is clear that our method has a better performance in terms of clutter suppression.

**Figure 12:**
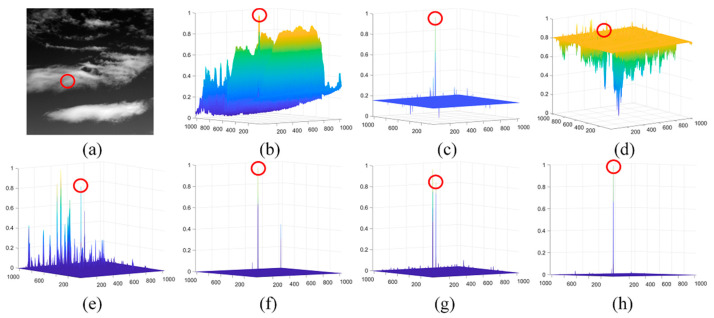
The detection results of different methods on S3: (**a**) the 1st image in S3 and (**b**–**h**) normalized 3-D mesh obtained by the LCM [18], IPI [10], MCPM [19], LIG [22], PSTNN [12], FKRW [15], and the proposed method, respectively.

The S3 sequence was acquired by our MWIR camera in good weather conditions with a small aircraft and with low cloud intensity. The results obtained by different methods are shown in Figure 12. The IPI and PSTNN, which are based on sparse representation and low-rank decomposition, have remarkable advantages over the LCM, MPCM, and LIG in terms of clutter suppression. The FKRW suppresses most of the clutter effectively, but still regards a small cloud as the target. The proposed method works well on S3, beating the other methods with a large margin.

**Figure 13:**
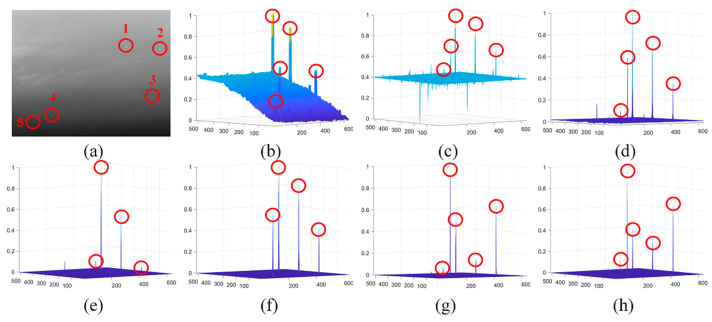
The detection results of different methods on S4: (**a**) the 79th image in S4 and (**b**–**h**) normalized 3-D mesh obtained by the LCM [18], IPI [10], MCPM [19], LIG [22], PSTNN [12], FKRW [15], and the proposed method, respectively.

The S4 sequence was acquired by our LWIR camera at night, and the number of the birds in S4 ranges from 3 to 5. The bird (target 5) in the lower left corner has small scale and quite low intensity, making it difficult to detect. The results in Figure 13 demonstrated that most of the methods have good detection performance on targets 1 to 4, and only the FKRW and the proposed method can distinguish the target 5 from the clutters effectively.

**Figure 14:**
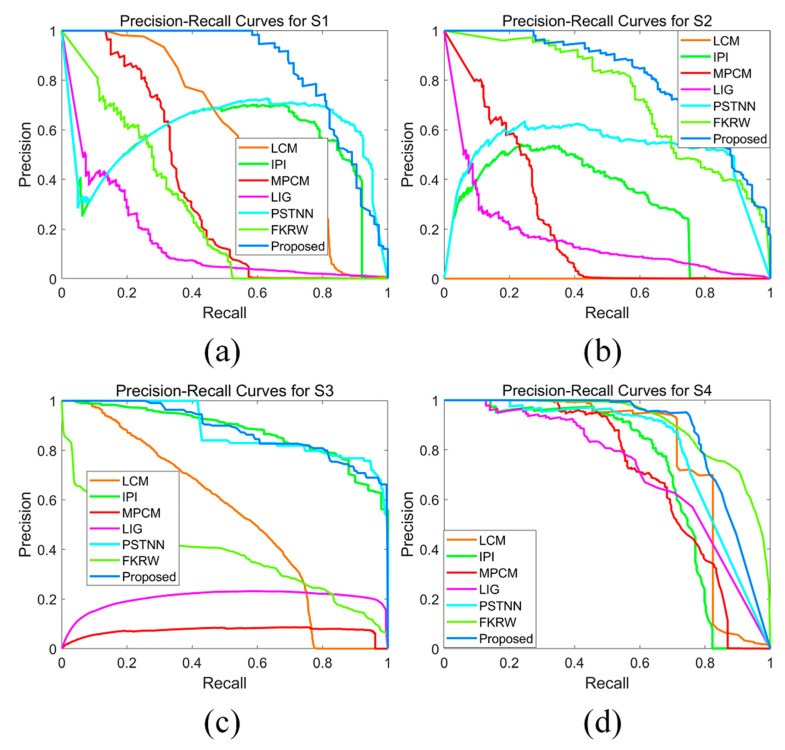
The PRC on different sequences: (**a**) PRC for S1, (**b**) PRC for S2, (**c**) PRC for S3, and (**d**) PRC for S4.

The precision–recall curves of the four different sequences are shown in the Figure 14. Since the test datasets are challenging, the overall performance of noise-sensitive methods including LCM, MPCM, and LIG is poor, while the performance of the methods based on data structure such as IPI and PSTNN are not robust. It is clear that the proposed method yields the most robust performance on these sequences. Although the PRC of FKRW on S4 is a bit better than our method, FKRW cannot obtain reliable results on S1 and S3. Moreover, our method has a better performance in terms of clutter suppression.

The comparison of the area under the curve (AUC) among different methods is given in Table 2. Although our proposed method does not achieve the largest AUC on the S4 sequence, the overall performance is the most stable, and the mean AUC is at least 22.3% higher than other methods (calculated based on the results of PSTNN).

We calculate the average time for one frame in different sequences and the comparison of the running time among different methods is shown in Table 3. Although the running time of all methods increases rapidly with the increasement of the resolution, it is clear that our method is the fastest and maintains a faster speed as the resolution increases. The computational cost of IPI and PSTNN increased dramatically due to the substantial increase in matrix size, while the running time of the LCM, MPCM, LIG, and the proposed method has an approximately linear relationship with the resolution. The computational cost of the FKRW is highly related to the data structure, but it is still far from real-time processing.

### 3.4. Merits and Limitations

The proposed method achieved stable detection performances for small-scale targets on the data set under the sky background. The computational cost of the proposed method is much less than that of the other baseline methods due to the fast extraction by the DoG filter. Moreover, the experimental results demonstrated that the combination of local contrast and gradient can effectively enhance small-scale targets and suppress background clutter. Comparing the results here with our earlier results in [23], the background clutter has been better suppressed at the expense of slightly more computational times.

However, the proposed method also has some limitations. First, missed detection can occur if the pre-processing stage cannot accurately extract the target due to the coarse-to-fine architecture. Second, the local contrast and gradient sometimes may not be good enough to meet the detection requirements of small and dim targets under all sky backgrounds, and it would be better if more local features can be utilized. Third, the proposed method cannot process the high-resolution image (1024×1024) in real-time.

## 4. Conclusions

In this paper, we proposed a novel and real-time method to detect a small and dim IR target in a sky background. The experimental results demonstrated that our proposed method achieves robust performance in terms of simultaneous target enhancement and background clutter suppression. More importantly, the proposed method runs much faster than the baseline methods.

Potential future directions include further improving the detection performance by utilizing more local features and accelerating the processing speed by introducing a parallel processing and pipeline architecture, which are essential for real-time processing of data in IR imaging systems.

## Figures and Tables

**Table 1:** The details of the test sequences.

Sequences	Frame Number	Resolution	Background Description	Target Characteristics
S1	100	320 × 256	Strong cloud	Aircraft with a size of 4 × 3
S2	100	320 × 256	Strong cloud with higher gray level	Dim aircraft with a size of 4 × 2
S3	50	1024 × 1024	Strong cloud with irregular shape	Aircraft with a size of 4 × 3
S4	50	640 × 512	Cloudy background	birds with size of around 3 × 3

**Table 2:** The comparison of the AUC among different methods.

		LCM	IPI	MPCM	LIG	PSTNN	FKRW	Proposed
Area Under Curve (AUC)	S1	0.5763	0.5442	0.3461	0.1493	0.5963	0.2678	**0.8532**
	S2	0.0001	0.3059	0.2168	0.1610	0.5058	0.7339	**0.8112**
	S3	0.5032	0.8780	0.0706	0.2014	0.8819	0.3773	**0.8835**
	S4	0.7892	0.7075	0.6716	0.7179	0.8082	**0.9050**	0.8678
	mean	0.4672	0.6089	0.3263	0.3074	0.6981	0.5710	**0.8539**

**Table 3:** The comparison of the running time among different methods.

		LCM	IPI	MPCM	LIG	PSTNN	FKRW	Proposed
Running time (s)	S1	0.035	9.227	0.048	1.726	0.028	0.141	**0.024**
	S2	0.038	7.913	0.047	1.702	0.027	0.075	**0.025**
	S3	0.546	854.3	0.734	24.52	2.153	1.201	**0.262**
	S4	0.141	374.6	0.201	7.386	0.108	0.152	**0.079**

## Data Availability

Data underlying the results presented in this paper are not publicly available at this time but may be obtained from the authors upon reasonable request.
